# Newtonian flow inside carbon nanotube with permeable boundary taking into account van der Waals forces

**DOI:** 10.1038/s41598-019-48614-2

**Published:** 2019-08-20

**Authors:** Yue Chan, Shern-Long Lee, Wenjian Chen, Lian Zheng, Yong Shi, Yong Ren

**Affiliations:** 1 Institute for Advanced Study, Shenzhen University, Nanshan District Shenzhen, Guangdong, 518060 China; 2 Department of Mechanical, Materials and Manufacturing Engineering, Faculty of Science and Engineering, The University of Nottingham Ningbo China, 199 Taikang East Road, Ningbo, China

**Keywords:** Applied mathematics, Coarse-grained models

## Abstract

Here, water flow inside large radii semi-infinite carbon nanotubes is investigated. Permeable wall taking into account the molecular interactions between water and a nanotube, and the slip boundary condition will be considered. Furthermore, interactions among molecules are approximated by the continuum approximation. Incompressible and Newtonian fluid is assumed, and the Navier-Stokes equations, after certain assumptions, transformations and derivations, can be reduced into two first integral equations. In conjunction with the asymptotic expansion technique, we are able to derive the radial and axial velocities analytically, capturing the effect of the water leakage, where both mild and exceptionally large leakages will be considered. The radial velocity obeys the prescribed boundary condition at the (im)permeable wall. Through the mean of the radial forces, the sufficiently large leakages will enhance the radial velocity at the center of the tube. On the other hand, unlike the classical laminar flow, the axial velocity attains its maximum at the wall due to the coupling effect with the radial forces as water is being pushed into the proximity of the inner wall. In addition, the axial velocity and the flux with the consideration of the suck-in forces, induced by the tubes’ entry turn out to be one order higher than that without the suck-in forces. All the aforementioned considerations might partially resolve the mysteriously high water penetration through nanotubes. Axial velocity also drops with the tube’s length when the water leakage is permitted and the suck-in forces will ease the decline rate of the axial velocity. The present mathematical framework can be directly employed into the water flow inside other porous nano-materials, where large water leakage is permitted and therefore are of huge practical impact on ultra-filtration and environmental protection.

## Introduction

Nanotechnology has dramatically reshaped almost every aspect in our modern life mainly due to the superior mechanical, electronic, magnetic and optical properties of nanomaterials^1^. Certain nanomaterials have been found to provide important means to purify wastewater and desalinate seawater, which are particularly vital owing to the everlasting economic growth and the prevailing global warming. Various methodologies namely adsorption, distillation, reverse osmosis and solar evaporation have been used to extract pure water^2,3^. However, most of the aforementioned methodologies are either energetically unfavor or are too costly to operate and maintain. Few of them are not even capable of removing pollutants effectively^2^. Polymer membrane embedding well-aligned carbon nanotubes can provide efficient channels for water transport, purification and desalination^4^ due to the following merits: (I) Nanotubes with appropriate pore sizes can generate right energy barriers at the entry of nanotubes in order to remove salt ions and only accept water into the tubes^5–8^; (II) Functionalized nanotubes are able to selectively accept or reject targeted ions mimicking biological channels^9–11^. Spinning carbon nanotubes can also generate the centrifugal force so as to facilitate better desalination effect^12^; (III) Carbon nanotubes possess self-cleaning, antifouling and reusable functionalities and hence they can be recycled leading to a low operational cost^2,13^; (IV) Due to the nearly frictionless and the hydrophobic inner surface of nanotubes, nanotubes can exhibit remarkably high water permeability^14–17^. In particular, the volumetric flow rate for carbon nanotube membranes can reach several orders of magnitude higher than that predicted by the continuum hydrodynamics theory and conventional fluid theory^16^; (V) The speed of an individual water molecule in a small radii nanotube could reach as high as 1000 m/s^18^. Therefore, the high water permeability and species selectivity offered by nanotubes turn them into a promising candidate for pollutant removal and water-ion separation.

Even numerous experiments and molecular dynamics simulations^19–21^ predict the rapid water transport inside nanotubes, effective mechanical approach for tackling such problem appears to be missing in the current literature. Single file transport of molecules are reported for ultra-small radii nanotubes^7,22^ so that continuum mechanics fail to address water flow inside such nanotubes. Therefore, herein, we only consider large radii nanotubes, in particular nanotubes with radius 20 Å, where the usage of continuum mechanics is eligible. Prakash and Yeom^23^ point out that for a confined nanoscale transport, if the size of species is 10 times smaller than the system dimension, the continuum approach is found to be approximately valid. For example, Majumder *et al*.^16^ adopt the continuum hydrodynamics theory to study the water flow inside nanotubes. However, the results are derived from thermal physics, which do not appeal to general readers. Popadic *et al*.^24^ adopt the Navier-Stokes equations with the partial-slip boundary conditions, which are extracted from molecular dynamics simulations to simulate water flow in carbon nanotube membrane. They predict the pressure losses at the nanotube’s entry and the enhancement of the volumetric flow rate in the nanotubes. However, analytical solutions are still absent since they adopt the finite volume to solve the problem. Continuum mechanics has also been used to predict the water flow inside nanotubes^14,15,25,26^, however they ignore the molecular effects. They simply incorporate the effect of slip boundary condition into the conventional Poiseuille flow model in order to predict rapid flow rate. To tackle such problem, Shaat and Zheng^27^ use a hybrid continuum-molecular mechanics to depict the fluidity and phase transitions of water in hydrophobic and hydrophilic nanotubes. However, their results rely heavily on the outputs of MD simulations. It is worthy to notice that a number of analytical solutions for Poiseuille flow has been derived in both cylindrical^28–30^ and rectangular porous channels^31,32^. To the best of the authors’ knowledge, high flow rate at the nanoscale has not been properly addressed using a mechanical approach.

To elucidate such a high water flow rate, without loss of generality, we merely scrutinize water flow in a carbon nanotube with radius 20 Å. Navier-Stokes (NS) equations are the best model to describe the fluid flow inside large radii nanotubes^25^. Even we expect that the fluid density and viscosity are not constant inside nanotube^33^, Newtonian fluid is still used here in order to avoid the unnecessary mathematical complexity. Despite the simple Newtonian fluid is assumed, our numerical results still reveal a boundary layer near the tube’s wall featuring variable water density inside nanotubes. As usual, we consider the slip boundary condition (See Eq. (2c)), however we also incorporate the molecular interactions, in particular the van der Waals forces, between water and the nanotube so as to capture the nano effect caused by the nanotube. Such molecular effects can be modeled by the continuum approximation^34,35^, which is exceptionally successful in tackling certain nano problems such as particle-laden flow inside nano-materials^36,37^, ultra-filtration/desalination^5,6,38,39^, and hydrogen production^10^ and storage using nanomaterials^40^, to name just a few. The present authors have initiated the preliminary study for the present problem, where only the impermeable boundary is considered^41^. In^41^, we find that the axial and radial velocities satisfy the prescribed boundary conditions. For a given value of the slip length, and upon incorporating the molecular interactions, the water flow rate has been lifted by almost seventh fold without considering such interactions. In this paper, we will consider permeable boundary conditions, where water leakage could be allowed probably due to some natural causes or material defects^25,42^. We comment that even the situation of permeable wall is rather fictitious for the case of nanotubes, however it might be our interest to know the effect for different levels of water leakage on the water flow inside nanotubes. Most importantly, the technique developed here can be employed to investigate water flow for other porous nano-materials, where permeable surfaces are prevailing. We rediscover the results derived from merely considering the impermeable wall, and more peculiar outcomes are also discovered for the axial and radial velocities when the water leakage is considered. Unlike other similar works, we try as much as possible to use applied mathematical analysis and mechanical approaches to describe the water flow inside large radii nanotubes.

## Theoretical Background

In this section, basic theoretical backgrounds are presented for the current problem. This section comprises two subsections, where the analytical solutions for the axial and radial velocities are derived in the first subsection, followed by the derivation of the radial forces and the suck-in forces generated by the carbon nanotube using the combination of continuum approximation, statistical mechanics and fluctuation-dissipation theorem in the second subsections. We comment that besides the aforementioned radial and suck-in forces, in reality, water entering a nanotube also suffers from the reduction in the entropy by forming well-ordered water, especially near the wall. Moreover, the formation of capillary meniscus in the beginning process of wetting costs energy too. However, we ignore such effects due to the sufficiently large radii of nanotubes and the continuum assumption of the water flow so that those effects can be compressed. Besides, only semi-infinite carbon nanotubes are considered so that we can only consider the effect from nanotube’s entry. Here, we incorporate the radial forces and the suck-in forces into the body acceleration and pressure terms of the Navier-Stokes equations, respectively in order to depict the fluid flow inside large radii nanotubes.

### Radial and axial velocities

**Figure 1:**
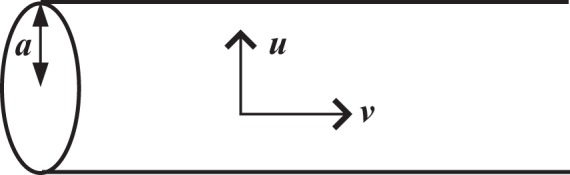
Schematic of the mathematical model.

Assuming that water is passing into a carbon nanotube with the axial velocity *v* and the radial velocity *u* (See Fig. 1 for details). The time independent Navier-Stokes equations and the incompressible condition are given by

1 $$\begin{array}{llll}u\frac{\partial u}{\partial r}+v\frac{\partial u}{\partial z} & = & -\frac{1}{\rho }\frac{\partial P}{\partial r}+\frac{\mu }{\rho }({\nabla }^{2}u-\frac{u}{{r}^{2}})+{g}_{r}, & \,(a)\\ u\frac{\partial v}{\partial r}+v\frac{\partial v}{\partial z} & = & -\frac{1}{\rho }\frac{\partial P}{\partial z}+\frac{\mu }{\rho }{\nabla }^{2}v, & \,(b)\\ \frac{\partial u}{\partial r}+\frac{u}{r}+\frac{\partial v}{\partial z} & = & \mathrm{0,} & \,(c)\end{array}$$

where *ρ*, *μ*, *P* and *g*_*r*_ are the water density, the dynamics viscosity, the pressure and the radial body acceleration, respectively. Even the gravitational force can be neglected here, we will reveal later that the radial forces generated by the nanotube on water can be inserted into the radial acceleration in Eq. (1).

As usual, the linear slip boundary condition is applied for *v*, which we will find later that, can be determined from microscopic information as shown in Eq. (27). In conjunction with the boundary conditions for the radial velocity *u*, we have

2 $$\begin{array}{llll}u(a,z) & = & b, & \,(a)\\ u\mathrm{(0,}z) & = & \mathrm{0,} & \,(b)\\ v(a,z) & = & -\ell \frac{\partial v}{\partial r}{|}_{r=a}, & \,(c)\end{array}$$

where *a* and $\ell$ are the tube’s radius and the usual slip length, respectively. In addition, *b* describes the constant water outflow from the nanotube’s wall. The second boundary condition is zero for the impermeable wall and tiny leakages. We emphasize here that we do not aim to provide a general class of solutions for Eqs (1) and (2). Due to the confinement of nanotubes, we focus on how to interwind molecular interactions with the nonlinear NS equations and then discover certain mechanical and physical phenomenon upon solving those equations.

Upon assuming *u* to be homogeneous along the *z*-direction, i.e. depends only on *r*, using the incompressibility condition, i.e. Eq. (1c) gives the general expression for the axial velocity: *v* = *v*_1_(*r*)*z* + *v*_0_(*r*) for some arbitrary functions *v*_0_(*r*) and *v*_1_(*r*). It is commonly perceived that the pressure *P* is only a function of *z*^21,25^, using *v* = *v*_1_(*r*)*z* + *v*_0_(*r*), Eq. (1a,b) become

3 $$-\rho {G}_{r}=\mu (\frac{du}{dr}+\frac{u}{r})-\frac{\rho }{2}{u}^{2},$$

4 $$u\frac{\partial v}{\partial r}+v\frac{\partial v}{\partial z}=\frac{-1}{\rho }\frac{dP}{dz}+\frac{\mu }{\rho }(\frac{{\partial }^{2}v}{\partial {r}^{2}}+\frac{1}{r}\frac{\partial v}{\partial r}),$$

where *G*_*r*_ denotes the usual anti-derivative of *g*_*r*_. Now, we make *u* and *v* in terms of the stream function *ψ*, that is

5 $$u=\frac{1}{r}\frac{\partial \psi }{\partial z}\,\,\,v=-\,\frac{1}{r}\frac{\partial \psi }{\partial r}.$$

As *u* is only a function of *r*, upon using Eq. (5), *ψ* becomes

6 $$\psi (r,z)=zf(r)+h(r),$$

for some arbitrary functions *f*(*r*) and *h*(*r*). Owing to the symmetry of the nanotube, it is more convenient to introduce the following change of variables, i.e. *ξ* = *a*^2^ − *r*^2^, where *ξ* ∈ [0, *a*^2^]. There are two reasons to make such transformation. Firstly, *ξ* occurs in solutions of the most laminar flows; Secondly, it converts the radial term from the denominator into the numerator so that the subsequent mathematics is easier to deal with. Upon using Eq. (5), *u* and *v* of the new variable *ξ* are given by

7 $$u(\xi )=\frac{F(\xi )}{\sqrt{{a}^{2}-\xi }},\,v(\xi ,z)=\mathrm{2[}zF^{\prime} (\xi )+H^{\prime} (\xi )],$$

where *F*(*ξ*) = *f*(*r*(*ξ*)) and *H*(*ξ*) = *h*(*r*(*ξ*)). In addition, ′ denotes the derivative with respect to *ξ*. It is clear to observe that we can determine *u* and *v* when both *F*(*ξ*) and *H*(*ξ*) are obtained. To this end, we substitute the current form of *u* and *v* into Eqs (3) and (4) to obtain the following coupled ordinary differential equations

8 $$-\rho {G}_{r}(\xi )=-\,2\mu F^{\prime} -\frac{\rho }{\mathrm{2(}{a}^{2}-\xi )}{F}^{2},$$

9 $$\begin{array}{ccc}\frac{1}{\rho }\frac{dP}{dz} = \{8\nu [({a}^{2}-\xi )F''' -F'']+4(FF''-{F'}^{2})\}z+\\\{8\nu [({a}^{2}-\xi )H'''-H'']+4(FH''-F' H' )\},\\ = {c}_{1}z+{c}_{2},\end{array}$$

where the kinematic viscosity denotes *ν* = *μ*/*ρ*. Intriguingly, *c*_1_ and *c*_2_ define the integral equations, which can be further made into

10 $$\frac{-{c}_{1}}{16}=\frac{F{^{\prime} }^{2}}{2}-\frac{d}{d\xi }\{\frac{\nu }{2}({a}^{2}-\xi )F^{\prime\prime} +\frac{1}{4}FF^{\prime} \},$$

11 $$\frac{-{c}_{2}}{4}=\frac{F^{\prime} H^{\prime} }{2}-\frac{d}{d\xi }\{\frac{\nu }{2}({a}^{2}-\xi )H^{\prime\prime} +\frac{FH^{\prime} }{4}\}\mathrm{.}$$

We notice that *c*_1_ and *c*_2_ define the “conservation rules” for *F*, and (*F* and *H*), respectively. With the aim of both first integrals, we can eventually solve *F* and *H*.

We observe from Eq. (7) that *F*′ = 0 for the case of the impermeable wall in order to avoid the divergence of *v* as *z* → ∞^41^, whereas *F*′ can be nonzero if water is allowed to enter or leave from the tube’s wall. Taking into account the permeable and slip boundary conditions, we propose the following regular asymptotic expansion for *F*(*ξ*), i.e.

12 $$F(\xi )={F}_{0}(\xi )+\varepsilon {F}_{1}(\xi )+{\varepsilon }^{2}{F}_{2}(\xi )+\mathrm{...}$$

Upon substituting *F*(*ξ*) into Eq. (9) to obtain

13 $$\begin{array}{rcl}-\rho {G}_{r}(\xi ) & = & -2\mu \{{F^{\prime} }_{0}(\xi )+\varepsilon {F^{\prime} }_{1}(\xi )+{\varepsilon }^{2}{F^{\prime} }_{2}(\xi )+\mathrm{...}\}\\  &  & -\frac{\rho }{\mathrm{2(}{a}^{2}-\xi )}{\{{F}_{0}(\xi )+\varepsilon {F}_{1}(\xi )+{\varepsilon }^{2}{F}_{2}(\xi )+\mathrm{...}\}}^{2}\mathrm{.}\end{array}$$

The zero order term produces ${F}_{0}(\xi )=\sqrt{\mathrm{2(}{a}^{2}-\xi ){G}_{r}(\xi )}$, where _0_ denotes the results owing to the impermeable wall, which is equivalent to *F*′_0_(*ξ*) = 0. The first order term yields $2\nu {F^{\prime} }_{1}(\xi )+\frac{\rho }{{a}^{2}-\xi }{F}_{0}(\xi ){F}_{1}(\xi )=0$, which relates *F*_1_(*ξ*) and *F*_0_(*ξ*). From which, we obtain

14 $${F^{\prime} }_{1}(\xi )=-\,\frac{1}{\nu }\sqrt{\frac{{G}_{r}(\xi )}{\mathrm{2(}{a}^{2}-\xi )}}{F}_{1}(\xi \mathrm{).}$$

We can deduce *F*_*n*_(*ξ*) inductively for higher order term. However, it turns out that the first order perturbation is sufficient to capture the permeable boundary condition as given in Eq. (2(a)). It is also worthy to comment that if the permeable condition is not a constant but some continuous functions, we can still use the Weierstrass approximation theorem^43^ to approximate such continuous functions by polynomials. In this case, higher order terms must be considered. To proceed, firstly, we deduce the radial velocity

15 $$u(\xi )=\frac{F(\xi )}{\sqrt{{a}^{2}-\xi }}=\frac{{F}_{0}(\xi )+\varepsilon {F}_{1}(\xi )}{\sqrt{{a}^{2}-\xi }}\mathrm{.}$$

Now, we need to fit this with the permeable boundary condition. For *u* at the wall, we obtain $u\mathrm{(0)}=\frac{\varepsilon }{|a|}{F}_{1}\mathrm{(0)}\equiv b$. Without loss of generality, we can let *ε* = *a* so that *F*_1_(*ξ*) immediately captures the inhomogeneous boundary condition (Eq. (2)(a)). Upon solving Eq. (14), we obtain

16 $${F}_{1}(\xi )=b{e}^{-\frac{1}{\sqrt{2}\nu }{\int }_{c}^{\xi }\sqrt{\frac{{G}_{r}(\tilde{\xi })}{{a}^{2}-\tilde{\xi }}}d\tilde{\xi }}\mathrm{.}$$

We comment that *c* is merely a number such that the antiderivative at *c* is zero. With the help of Eq. (15), and using *F*_0_ and *F*_1_, we deduce the expression for the radial velocity

17 $$u(\xi )=\sqrt{2{G}_{r}(\xi )}+\frac{ab}{\sqrt{{a}^{2}-\xi }}{e}^{-\frac{1}{\sqrt{2}\nu }{\int }_{c}^{\xi }\sqrt{\frac{{G}_{r}(\tilde{\xi })}{{a}^{2}-\tilde{\xi }}}d\tilde{\xi }}.$$

We comment that while the first term represents the radial velocity induced by the radial forces of the nanotube, the second term couples the leaking velocity, *b* with the radial forces. Now, we remain to obtain the axial velocity *v*(*ξ*). From Eq. (7), we can determine the axial velocity *v*(*ξ*), which is given by

18 $$\begin{array}{rcl}v(\xi ) & = & 2\{zF^{\prime} (\xi )+H^{\prime} (\xi )\},\\  & = & -\{\frac{ab}{\nu }\sqrt{\frac{2{G}_{r}(\xi )}{{a}^{2}-\xi }}{e}^{-\frac{1}{\sqrt{2}\nu }{\int }_{c}^{\xi }\sqrt{\frac{{G}_{r}(\tilde{\xi })}{{a}^{2}-\tilde{\xi }}}d\tilde{\xi }}\}z+2H^{\prime} (\xi \mathrm{).}\end{array}$$

To reach the final line of Eq. (18), we once again use *F*_0_′(*ξ*) = 0, which is a condition for the impermeable wall. To finalize our calculation, we have to compute *H*′(*ξ*). Fortunately, the first integral derived in Eq. (11) provides an effective mean to determine *H*′(*ξ*), at least perturbatively. Upon letting *K*(*ξ*) = *H*′(*ξ*) and using Eq. (11), we obtain

19 $$-\frac{{c}_{2}}{4}=\frac{a{F^{\prime} }_{1}K}{2}-\frac{d}{d\xi }\{\frac{\nu }{2}({a}^{2}-\xi )K^{\prime} +\frac{({F}_{0}+a{F}_{1})K}{4}\}\mathrm{.}$$

Analogous to *F*(*ξ*), we assume a regular perturbation expansion for *K*(*ξ*), i.e.

20 $$K=\mathop{\sum }\limits_{n\mathrm{=0}}^{\infty }{a}^{n}{K}_{n}={K}_{0}+a{K}_{1}+{a}^{2}{K}_{2}+\mathrm{....,}$$

where we have replaced the usual *ε* by the tube’s radius *a*, that has been discussed above and where *K*_*n*_ can be obtained by solving the following system of first order ordinary differential equations:

$$\frac{\nu }{2}\xi {K^{\prime} }_{0}-\frac{{F}_{0}}{4}{K}_{0}+{P}_{0}(\xi )=\mathrm{0,}$$

$$\frac{\nu }{2}\xi {K^{\prime} }_{1}-\frac{{F}_{0}}{4}{K}_{1}+{P}_{1}(\xi ,{K}_{0}(\xi ))=\mathrm{0,}$$

$$\frac{\nu }{2}\xi {K^{\prime} }_{n}-\frac{{F}_{0}}{4}{K}_{n}+{P}_{n}(\xi ,{K}_{0}(\xi ),\,\mathrm{...,}\,{K}_{n-1}(\xi ))=\mathrm{0,}\,\,n\ge \mathrm{2,}$$

where

21 $$\begin{array}{rcl}{P}_{0}(\xi ) & = & \frac{{{\bf{c}}}_{{\bf{2}}}}{4}\xi ,\\ {P}_{1}(\xi ,{K}_{0}(\xi )) & = & \frac{1}{2}{\int }_{c}^{\xi }\,{F^{\prime} }_{1}{K}_{0}d\tilde{\xi }-\frac{{F}_{1}{K}_{0}}{4},\\ {P}_{n}(\xi ,{K}_{0}(\xi ),\,\mathrm{...,}\,{K}_{n-1}(\xi )) & = & \frac{1}{2}{\int }_{c}^{\xi }\,{F^{\prime} }_{1}{K}_{n-1}d\tilde{\xi }-\frac{{F}_{1}{K}_{n-1}}{4}-\frac{\nu }{2}{K^{\prime} }_{n-2}\,\,n\ge 2.\end{array}$$

The solution to the above odes can be computed recursively, which is given by

22 $${K}_{n}={Q}^{-1}(\xi )\{-\frac{2}{\nu }{\int }_{c}^{\xi }\,\frac{{P}_{n}(\tilde{\xi },{K}_{0}(\tilde{\xi }),\,\mathrm{...,}\,{K}_{n-1}(\tilde{\xi }))Q(\tilde{\xi })}{\tilde{\xi }}d\tilde{\xi }\},$$

where the integration factor is $Q\,(\xi )={e}^{-\frac{1}{2\nu }{\int }_{c}^{\xi }\frac{{F}_{0}}{\tilde{\xi }}d\tilde{\xi }}$. Recap, we have the following expressions for the radial velocity *u*(*ξ*) and the axial velocity *v*(*ξ*):

23 $$\begin{array}{rcl}u(\xi ) & = & \sqrt{2{G}_{r}(\xi )}+\frac{ab}{\sqrt{{a}^{2}-\xi }}{e}^{-\frac{1}{\sqrt{2}\nu }{\int }_{c}^{\xi }\sqrt{\frac{{G}_{r}(\tilde{\xi })}{{a}^{2}-\tilde{\xi }}}d\tilde{\xi }},\\ v(\xi ) & = & 2\mathop{\sum }\limits_{n\mathrm{=0}}^{\infty }\,{a}^{n}{K}_{n}(\xi )-\{\frac{ab}{\nu }\sqrt{\frac{2{G}_{r}(\xi )}{({a}^{2}-\xi )}}{e}^{-\frac{1}{\sqrt{2}\nu }{\int }_{c}^{\xi }\sqrt{\frac{{G}_{r}(\tilde{\xi })}{{a}^{2}-\tilde{\xi }}}d\tilde{\xi }}\}z,\end{array}$$

where ${K}_{n}(\xi )={Q}^{-1}(\xi )\{-\frac{2}{\nu }{\int }_{c}^{\xi }\,\frac{{P}_{n}(\tilde{\xi },{K}_{0}(\tilde{\xi }\mathrm{),}\,\mathrm{...},{K}_{n-1}(\tilde{\xi }))Q(\tilde{\xi })}{\tilde{\xi }}d\tilde{\xi }\}$. We comment that while the radial velocity is completely driven by the radial forces and the leakage, the axial velocity interwinds the suck-in forces, the radial forces and the leakage. The above observations agree well with the massive radius-dependent flow slippage in carbon nanotubes^44^. In addition, the leakage determines how far water can penetrate through the nanotube. According to Eq. (23), we can easily deduce the maximum penetration length *L*_*p*_ of the present problem by letting *v*(*ξ*) = 0, which is given by

24 $${L}_{p}=\frac{2\mathop{\sum }\limits_{n\mathrm{=0}}^{\infty }\,{a}^{n}{K}_{n}}{(\frac{ab}{\nu })\sqrt{\frac{2{G}_{r}(\xi )}{({a}^{2}-\xi )}}{e}^{-\frac{1}{\sqrt{2}\nu }{\int }_{c}^{\xi }\sqrt{\frac{{G}_{r}(\tilde{\xi })}{{a}^{2}-\tilde{\xi }}}d\tilde{\xi }}}\mathrm{.}$$

Finally, we remain to determine the conservation constant *c*_2_ in order to compute *K*_0_ and hence *K*_*n*_, *n* ∈ *Z*^+^ (See the bolded letter **c**_**2**_ in Eq. (21)). This can be done by using the final boundary condition, i.e. Eq. (2c) and imposing no slip velocity at *L*_*p*_. From Eq. (2c), we deduce

25 $$v(\xi =\mathrm{0,}\,z)=2a\ell \frac{\partial v}{\partial \xi }{|}_{\xi =0},$$

which leads to

26 $$\begin{array}{c}2\mathop{\sum }\limits_{n\mathrm{=0}}^{\infty }\,{a}^{n}{K}_{n}\mathrm{(0)}-4a\ell \mathop{\sum }\limits_{n\mathrm{=0}}^{\infty }\,{a}^{n}{K^{\prime} }_{n}\mathrm{(0)}\\ =\,\{\frac{b\sqrt{2{G}_{r}\mathrm{(0)}}}{\nu }-\frac{\sqrt{2}b\ell }{a\nu \sqrt{{G}_{r}\mathrm{(0)}}}[{a}^{2}{G^{\prime} }_{r}\mathrm{(0)}+{G}_{r}\mathrm{(0)}]+\frac{2b\ell }{{\nu }^{2}}{G}_{r}\mathrm{(0)}\}\mathrm{Exp}\mathrm{(0)}z,\end{array}$$

where $\mathrm{Exp}\mathrm{(0)}={e}^{-\frac{1}{\sqrt{2}\nu }{\int }_{c}^{\xi =0}\sqrt{\frac{{G}_{r}(\tilde{\xi })}{{a}^{2}-\tilde{\xi }}}d\tilde{\xi }}$. Usually, the slip length is computed from performing molecular dynamics simulations^17^. Since the left hand side is a constant while the right hand side is a function of *z*, which is only possible if both sides are equal to zero leading to

27 $$\ell =\frac{\sqrt{2{G}_{r}\mathrm{(0)}}}{\frac{\sqrt{2}}{a\sqrt{{G}_{r}\mathrm{(0)}}}[{a}^{2}{G}_{r^{\prime} }\mathrm{(0)}+{G}_{r}\mathrm{(0)}]-\frac{2{G}_{r}\mathrm{(0)}}{\nu }}=\frac{1}{2a}{(\frac{d\mathrm{ln}K}{d\xi })}^{-1}{|}_{\xi =0}\mathrm{.}$$

We comment that the first and second equalities can be used to determine the slip length $\ell$ theoretically and the constant *c*_2_ numerically, respectively. The slip length is related to the radial forces and the pressure drop across the tube, which matches well with the result obtained from^21^.

### Molecular forces generated by nanotube and fluctuation dissipation theorem

Here, we determine the pressure induced by the entry of nanotube and the radial acceleration *g*_*r*_(*r*) generated by the molecular interactions in nanotube. It has been shown that when a water molecule reaches nanotube’s vicinity, due to the asymmetry of the tube’s entry, it will experience strong suck-in forces^34,35^. According to^10,38^, the total axial molecular forces acting on the water molecule when tunneling into the nanotube, *F*^*tot*^ can be written as

28 $${F}^{tot}={F}_{O-T}+{F}_{{H}_{1}-T}+{F}_{{H}_{2}-T}+{F}_{hydra},$$

where *F*_*O*−*T*_, ${F}_{{H}_{1}-T}$, ${F}_{{H}_{2}-T}$ are the molecular forces between the oxygen and the nanotube, between the first hydrogen on water molecule and the nanotube, between the second hydrogen and the nanotube, respectively. In addition, *F*_*hydra*_ denotes the hydraulic force.

Now, we temporarily ignore the hydraulic force but we will revisit it in the later part of the subsection. We employ the methodology as given in^34,35^, from which they adopt the continuum approximation to coarse grain all molecular forces. In addition, the analytical expression for each terms of Eq. (28) can be found in^5,6^. Water molecules undergo rapid at finite temperatures and hence we can employ Boltzmann’s statistics to derive the ensemble axial molecular forces, gives

29 $${F}_{z}(z)\,:={F}^{Avg}=\frac{{\sum }_{j}\{{F}_{j}^{tot}\exp (-\,\beta {V}_{j}^{tot})\}}{{\sum }_{j}\exp (-\,\beta {V}_{j}^{tot})},$$

where ${F}_{j}^{tot}$ and ${V}_{j}^{tot}$ denote the *j*-orientation of the total axial forces and the total energy possessing such forces, which can be easily obtained by integrating the total force, *F*^*tot*^ with respect to *z*, respectively. Besides, we assume point masses for the water molecules and model the interactions among the molecules using the Lennard-Jones potential^45^. Given that, the radial acceleration *g*_*r*_ reads

30 $${g}_{r}={m}^{-1}{\int }_{-\pi }^{\pi }\,{\int }_{-\infty }^{\infty }\,\{\frac{24\varepsilon }{\sigma }[2{(\frac{\sigma }{\rho })}^{13}-{(\frac{\sigma }{\rho })}^{7}]\}adzd\theta ,$$

where *ρ*, *ε* and *σ* denote the distance between molecules, the potential well depth and the van der Waals diameter, respectively. Further, *z* and *θ* denote the *z* length and the azimuthal coordinates in the usual cylindrical coordinate system, respectively. We comment that the radial acceleration acts radially on water in the nanotube and the analytical form reads

31 $${g}_{r}=24{m}^{-1}\varepsilon \{2{\sigma }^{12}{I}_{13}-{\sigma }^{6}{I}_{7}\},$$

where *ρ* = (*a*^2^ + *r*^2^ − 2*ar* cos *θ* + *z*^2^)^1/2^ and *r* is the radial coordinate. In addition, *I*_*n*_ is given by

$${I}_{n}={\int }_{-\pi }^{\pi }\,{\int }_{-\infty }^{\infty }\,\frac{a}{{\rho }^{n\mathrm{/2}}}d\theta dz=\frac{4\pi a}{{(a-r)}^{n-1}}\{{\int }_{0}^{\pi \mathrm{/2}}{\cos }^{n-2}\theta d\theta \}F(\frac{n-1}{2},\frac{1}{2};\,\mathrm{1;}\,\frac{-4ar}{{(a-r)}^{2}}),$$

where *F* denotes the standard hypergeometric function and *G*_*r*_(*r*) is defined by integrating *g*_*r*_ (See Eq. (4)) with respect to *r*.

**Figure 2:**
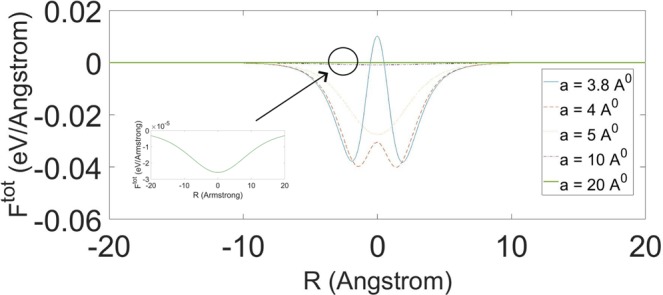
Total axial molecular forces *F*_*z*_(*z*) for water molecule intruding into nanotube of radius *a*.

Now, we take both the hydraulic and applied forces into account. Here, we only consider an approximate approach, which is derived from solving the Langevin equation. Firstly, we coarse grain the 3D motion of a water molecule into an one dimensional motion, which is particularly valid in the vicinity of nanotube’s entry. We further assume that the hydraulic force, *η*(*t*) forms a white noise with the average amplitude <*η*^2^> = 2*γT*, where *γ* and *T* denote the dissipation constant for the water molecule and the temperature, respectively. Given that, the Langevin equation can be written as

32 $$mz^{\prime\prime} +\gamma {\rm{\Theta }}(z)z^{\prime} ={F}_{z}(z)+{P}_{app}{\rm{\Omega }}+\eta (t){\rm{\Theta }}(z),$$

where *m*, *P*_*app*_ and Ω denote the mass for a water molecule, the external pressure and the surface area of the single water molecule, respectively. We comment that the step function Θ(*z*) is added taking into account that the water molecule experiences no friction and random force once it travels in the nanotube. We also comment that *F*_*z*_(*z*) can be determined in Eq. (29) and solve Eq. (32) numerically to obtain the acceleration and hence the suck-in forces. Readers can regard the suck-in forces as a combined effect from the ensemble axial forces, external pressure and the hydraulic force, which form an impulse at the tube’s entry (See Fig. 2 for reference).

## Results and Discussion

In this section, we derive some numerical results and make discussion. When water approaches the proximity of the nanotube’s entry, it will experience the suck-in forces. Upon using Eq. (29), the axial molecular forces *F*_*z*_(*z*) of a single water molecule tunneling into carbon nanotubes of radii 3.8, 4, 5, 10 and 20 Å are given, respectively in Fig. 2. We note that the parameters used can be found in^41^. Furthermore, the negative and positive forces represent the attractive and repulsive forces, respectively.

It is inconclusive to tell whether a water molecule can be sucked into an nanotube with radius 3.8 Å because both the positive and negative forces exist at the entry of the nanotube. However, after careful calculations, Chan and Hill^6^ have deduced that the water molecule can spontaneously get into the nanotube. They also show that water can transport into nanotubes with radii larger than 3.4 Å and the magnitude of the axial forces decrease with the tube’s radius. It is clear from Fig. 2 that these axial forces look like impulses at the tube’s entry for all the proposed tube’s radii. Therefore, those axial forces are only dominant at the tube’s entry and hence it can be deemed as the pressure as shown in Eq. (1a,b).

**Figure 3:**
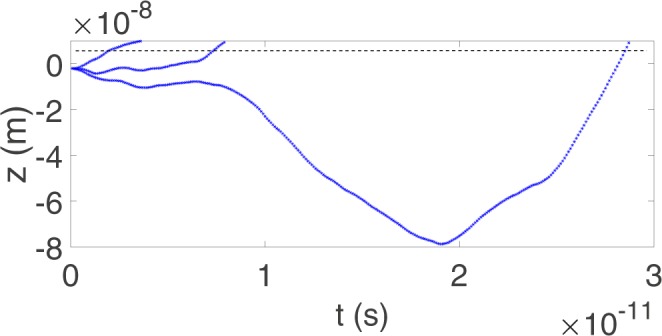
Monte-Carlo simulations for Eq. (32).

However, the water molecule will also experience the forces arising from the bulk solution and the applied external force. To capture such effects, we adopt the Langevin equation given in Eq. (32). Since *F*_*z*_(*z*) is highly nonlinear, unlike other classical linear functional forms for *F*_*z*_(*z*), where *F*_*z*_(*z*) is just a function of *z* so that simple Fourier analytical treatment can be adopted to solve the Langevin equation. Instead, we solve *F*_*z*_(*z*) numerically using a Monte-Carlo simulation. The numerical outputs help approximating the total pressure *P* exerted on water at the tube’s opening. We fix the radius of a nanotube 20 Å. Using *P*_*app*_ = 1 × 10^5^ Pa, *γ* = 1*e* − 9 kgs^−1^ and *T* = 300 K to carry the Monte-Carlo simulations and some few numerical results are shown in Fig. 3.

While the motion of the water molecule executes random motion outside the nanotube, it admits a more linear motion inside the tube. Accelerations can be extracted from the *t* − *z* graph and the pressure driven by the nanotube entry can then be estimated. We will also show later that such pressure taking into account the interactions among water molecules, the external pressure and the suck-in forces induced by the nanotube will enhance the axial velocity of water penetrating through the nanotube. Suk and Aluru^21^ also adopt the molecular dynamics simulations and show that the entrance effect enhances the water flow through carbon nanotubes.

**Figure 4:**
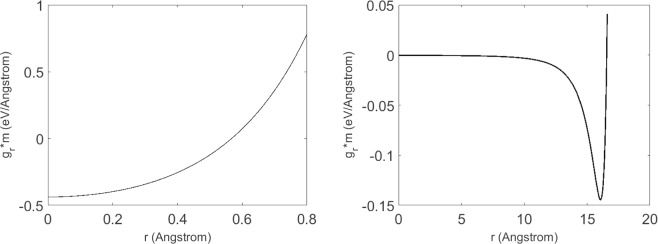
(Left) Radial force (m *g*_*r*_ for nanotube with radius 4 Å, where *m* denotes the mass of a water molecule); (Right) Radial force for nanotube with radius 20 Å.

Once water transports inside the nanotube, it will also experience the radial forces generated by the nanotube, which has been derived in Eq. (31). Numerical results of the radial forces, i.e. *mg*_*r*_, where *m* denotes the mass of a single water molecule, induced by nanotubes of radii 4 and 20 Å are shown in Fig. 4.

We observe that the radial forces are acting radially on all water in the nanotube and hence it is equivalent to the radial body acceleration, i.e. *g*_*r*_ in Eq. (1a). For the nanotube with radius 4 Å, the maximum radial force attains at the tube’s center. Obviously, this contradicts to the prescribed boundary condition, i.e. *u*(*r* = 0) = 0. However, for those ultra-small radii nanotubes, continuum assumption is broken and water molecules obey the single-file transport, which has been discussed in^7^. On contrary, for nanotube with radius 20 Å, the radial forces are zero at both the center and the proximity of wall, automatically satisfying the prescribed boundary conditions. We also comment that water molecules are unable to touch the nanotube wall as they are repulsed by the forces induced by the wall.

**Figure 5:**
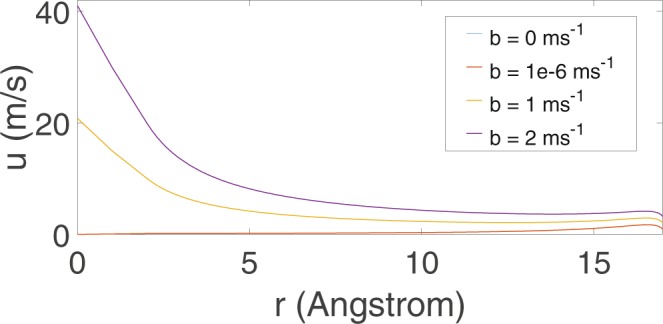
Radial velocities for nanotube of radius 20 Å for various *b* m/s.

Now we determine the radial velocity by adopting the following parameters as given in^25^, where tube radius (*a* = 20 Å), tube length (*L* = 10 Å), viscosity (*μ* = 10^−3^ Pa s) and density (10^3^ kg m^−3^). We also assume that *dP*/*dz* ≈ Δ*P*/*L* and the mechanical pressure drop across the tube is given by Δ*P*_*app*_ = 10^5^ Pa, and Δ*P* = Δ*P*_*app*_ + pressure induced by the tube’s entry and the bulk solution, where the average value can be extracted from Fig. 3 when we turn the applied pressure off to determine the flow in nanotubes with relatively large radii. The radial velocity is derived from Eq. (23) for various speeds of leakage *b*, which is shown in Fig. 5.

For the impermeable wall, the maximum radial velocity attains at *r* ≈ 16.8 Å, and the radial velocity is zero at both the tube’s wall and center, which corresponds well with the radial force as given in Fig. (4). It also fulfils the prescribed boundary conditions, i.e. Eq. (2a,b) by letting *b* = 0 m/s. For tiny *b*, in this case *b* = 1*e* − 6 m/s, the radial velocity almost collides with that of the impermeable wall and therefore, their flow characteristics are almost identical. For large *b*, however, the radial velocity shows a peculiar phenomenon, where it does not satisfy the prescribed boundary condition in Eq. (2b) due to the large leakage, with the aim of radial forces, induces the drag of water towards the tube’s wall through the conservation of linear momentum so that the maximum radial velocity occurs at the center of the tube.

**Figure 6:**
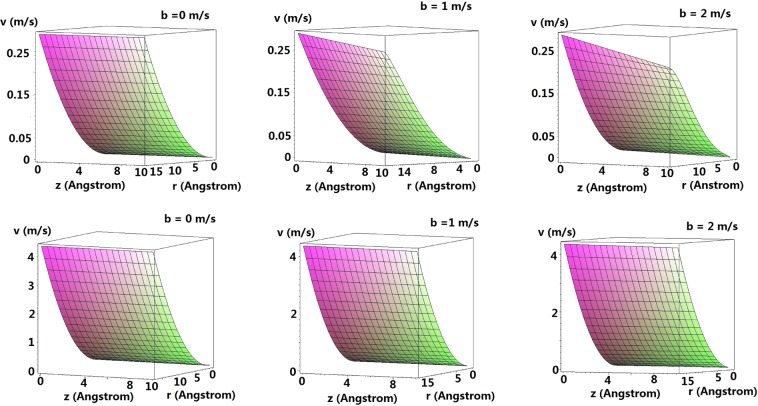
Axial velocities without (Upper) and with (Lower) consideration of suck-in forces for various *b* m/s.

Using Eq. (23), or else simply estimate *c*_2_ from using Eq. (9), the numerical result for the axial velocity with and without the consideration of suck-in forces for various *b* is shown in Fig. 6.

**Figure 7:**
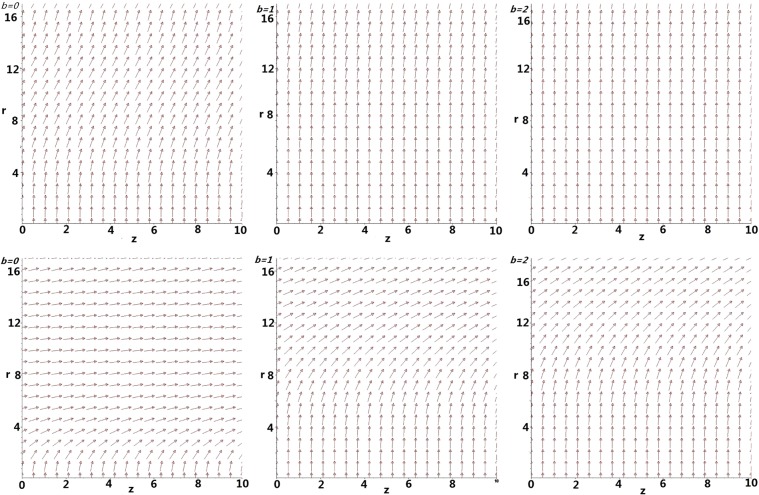
Vector fields without (Upper) and with (Lower) consideration of suck-in forces for various *b* m/s.

Unlike the classical parabolic flow, where the maximum velocity attains at the tube’s center. The axial velocity reaches its maximum value at the tube wall whereas the minimum value occurs at the center of the nanotube because of the effect arising from the radial forces (See the coupled ODE, i.e. Eqs (10) and (11) leading to the axial velocity which depends also on the radial forces, i.e. Eq. (23)). For all the proposed cases, we attribute such phenomenon as a result of water being pushed towards the wall’s vicinity (See Fig. 7), where it is more habitable to stay with (See Fig. 4) leading to the higher axial velocity over there. As the radius of the tube increases, such molecular effect will be neglectable resulting in classical parabolic flow. We also notice that the flow field is similar to that derived from Rayleigh’s equation, where the velocity is at rest on the water surface whereas the velocity attains its maximum on the the bottom^43^. It is clear that the axial velocity does not drop with *z* for *b* = 0 m/s with and without the consideration of suck-in forces. However, for large *b*, the axial velocity drops significantly with *z* and drops more severely for larger *b*. Besides, the decline of the axial velocity is more promising without the consideration of suck-in forces due to the longer interactions of water molecules with the tube’s wall. Most importantly, the strength of the axial velocity with the consideration of suck-in forces is in generally one order higher than that without the suck in forces, which might partially solve the mysteriously high water permeability inside nanotubes.

Now, we apply both the radial and axial velocities to produce field plots, which are shown in Fig. 7, where the location of the subplots for Fig. 7 corresponds exactly with that of Fig. 6.

For *b* = 0 m/s, while the radial velocity is more dominant for the case of no suck-in forces, the axial velocity is more significant in the presence of suck-in forces. In addition, the higher axial velocity induced by the suck-in forces pushes less water onto the nanotube’s wall; For large *b*, the radial velocity is again more dominant for the case without the suck-in forces and becomes more significant for larger *b*, whereas the axial velocity is more dominant for the case of the suck-in forces. For the both cases, radial velocity is higher in the center than that on the tube’s wall. Moreover, with the consideration of suck-in forces, the higher the speed of leakage, more water is being pushed onto the tube’s wall and hence producing the thinner “boundary layer”. It is also clear from Eq. (24) that the larger *b* leads to the shorter penetration length.

**Figure 8:**
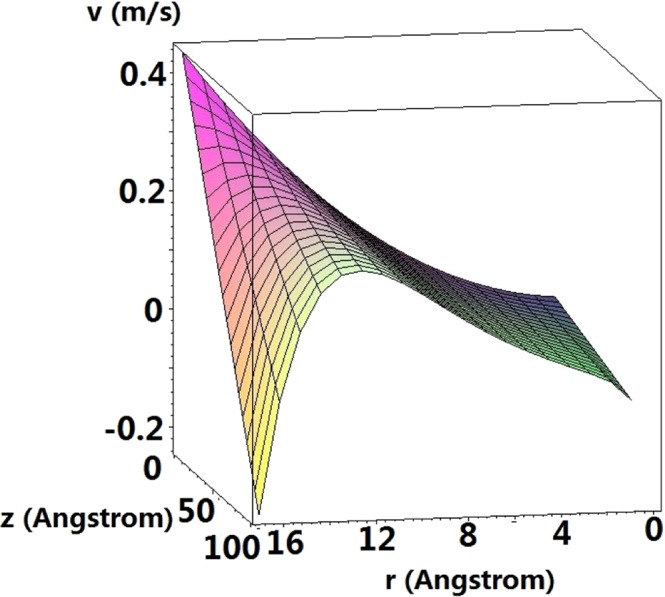
Axial velocity for nanotube of length 100 Å, where the suck-in forces are considered and *b* = 2 m/s.

Now we extend the length of nanotube into 100 Å so that the exhaustion of water with the tube’s length can be reached (See *L*_*p*_ in Eq. (24) and Fig. 8). Above which, the axial velocity is negative and the numerical results are voided. We observed from Fig. 8 that such length depends on the radius and *L*_*p*_ approaches faster in the center and the boundary of the nanotube than the rest of the nanotube. In other words, when the water is empty at the tube’s wall and center, there still exists some residual flow inside the nanotube.

The axial flux can be written by **J**(*r*, *z*) = *ρ***v**(*r*, *z*) so that the average axial flux can be computed using $< {\bf{J}} > =(\rho /V){\int }_{0}^{L}\,{\int }_{S}\,{\bf{v}}(r,z)\cdot {\bf{dS}}dz$, where *V*, **dS** and *dz* denote the volume of the hallow nanotube, the surface element and the length element of nanotube, respectively. The average axial flux for *b* = 0, 1, 2 m/s with and without the suck-in forces is given in Table 1.

**Table 1:** Average flux for various *b* with and without suck-in forces.

*J*_*avg*_ (kgm^−2^ s^−1^)	*b* = 0	*b* = 1*e* − 6	*b* = 1	*b* = 2
Without suck-in forces	200.0000000	199.9999941	194.4420575	189.5248248
With suck-in forces	3021.583478	3021.583470	3015.741997	3009.939719

The average axial flux for the case of suck-in forces is one order higher than that without the suck-in forces. Besides, due to the leakage, the average flux decreases when *b* increases for both cases. The relative decrease is larger without the suck-in forces than that with the suck-in forces due to the longer interactions of water with the tube’s wall. We also notice that even though there is no quality difference between *b* = 0 m/s and *b* = 1*e* − 6 m/s, we can observe tiny difference between the fluxes for both cases.

Last but not least, we comment on the correctness of the slip length given in Eq. (27) by comparing the present result with the linearized solution for *b* = 0 m/s derived in^41^, which is given by

33 $$v(r)=-\frac{{\rm{\Delta }}P}{\mu L}({a}^{2}-{r}^{2})+\frac{2a\ell {\rm{\Delta }}P}{\mu L}\mathrm{.}$$

For *b* = 0 m/s without the consideration of suck-in forces, upon inserting the value of the slip length computed using Eq. (27) into Eq. (33), we obtain *v*(*r*) = 0.8 + 0.001*r*^2^, where *r* is in Å. The outcome is in the same order with the result derived using Eq. (18) (See the upper figure of Fig. 6 with *b* = 0 m/s. The discrepancy is mainly due to the over-simplification of linearization. Even Eq. (33) appears to involve no microscopic information, it has already been absorbed in Δ*P* and the slip length $\ell$.

## Conclusion

In conclusion, Newtonian fluid with the slip boundary condition is used to simulate the fluid flow inside carbon nanotube with the permeable wall. Molecular effect is considered, and both the radial and axial velocities are derived analytically. There is no quality difference for flow characteristics between the impermeable wall and when the leakage outflow is tiny. While the large leakages induce the higher radial velocity at the center of nanotube, and the axial velocity and average axial flux with the consideration of suck-in forces are almost one order higher than that without suck-in forces. These might partially explain the mystery of unexpectedly high flow rate occurring inside nanotubes. The present paper also open up a new mathematical and mechanical approach to describe water flow inside other porous nano-materials, where highly permeable surfaces are allowed.
